# Monitoring Group Activity of Hamsters and Mice as a Novel Tool to Evaluate COVID-19 Progression, Convalescence, and rVSV-ΔG-Spike Vaccination Efficacy

**DOI:** 10.3389/fbioe.2021.737627

**Published:** 2021-10-01

**Authors:** Sharon Melamed, Boaz Politi, Ettie Grauer, Hagit Achdout, Moshe Aftalion, David Gur, Hadas Tamir, Yfat Yahalom-Ronen, Shlomy Maimon, Efi Yitzhak, Shay Weiss, Amir Rosner, Noam Erez, Shmuel Yitzhaki, Shmuel C Shapira, Nir Paran, Emanuelle Mamroud, Yaron Vagima, Tomer Israely

**Affiliations:** Israel Institute for Biological Research (IIBR), Ness-Ziona, Israel

**Keywords:** SARS-CoV-2, COVID-19, rVSV-ΔG-spike, animal group activity, vaccine

## Abstract

The COVID-19 pandemic initiated a worldwide race toward the development of treatments and vaccines. Small animal models included the Syrian golden hamster and the K18-hACE2 mice infected with SARS-CoV-2 to display a disease state with some aspects of human COVID-19. A group activity of animals in their home cage continuously monitored by the HCMS100 (Home cage Monitoring System 100) was used as a sensitive marker of disease, successfully detecting morbidity symptoms of SARS-CoV-2 infection in hamsters and in K18-hACE2 mice. COVID-19 convalescent hamsters rechallenged with SARS-CoV-2 exhibited minor reduction in group activity compared to naive hamsters. To evaluate the rVSV-ΔG-spike vaccination efficacy against SARS-CoV-2, we used the HCMS100 to monitor the group activity of hamsters in their home cage. A single-dose rVSV-ΔG-spike vaccination of the immunized group showed a faster recovery than the nonimmunized infected hamsters, substantiating the efficacy of rVSV-ΔG-spike vaccine. HCMS100 offers nonintrusive, hands-free monitoring of a number of home cages of hamsters or mice modeling COVID-19.

## Introduction

The coronavirus disease 2019 (COVID-19) pandemic initiated a worldwide race toward the development of treatments and vaccines against the emerged RNA virus severe acute respiratory syndrome corona virus-2 (SARS-CoV-2). Early on, the research focused on searching and defining an appropriate animal model able to represent disease symptoms and progression patterns of SARS-CoV-2–infected humans, to be used in the preclinical studies (Cohen, 2020). During earlier coronavirus outbreaks of severe acute respiratory syndrome (SARS-CoV-1) in 2002 and Mediterranean respiratory syndrome (MERS-CoV) in 2012, golden Syrian hamsters infected with various SARS-CoV strains were implemented to show viral replication and lung pathology (Roberts et al., 2008). At the present SARS-CoV-2 outbreak, hamsters were found to be an appropriate animal model of COVID-19 mainly based on the demonstration of viral replication and pathological damage of the nasal and lung tissues associated with a high viral load. Their clinical symptoms however were subtle and mainly relied on weight loss (Chan et al., 2020; Yuan et al., 2021).

The specificity of the SARS-CoV-2 virus to the human angiotensin I–converting enzyme 2 (hACE2) was found to be a significant barrier in developing animal models. Mice abundantly used in pharmacological and immunological studies neither support SARS-CoV-2 infection nor exhibit any signs of morbidity following infection (Cohen, 2020). Consequently, a SARS-CoV-2 transgenic mouse model (K18-hACE2) was developed by inserting the hACE2 gene into the mouse genome (Yang et al., 2007).

Recently, a recombinant replication competent VSV-∆G-spike vaccine (rVSV-∆G-spike) was developed, in which the glycoprotein of VSV was replaced by the spike protein of SARS-CoV-2. A single-dose vaccination of hamsters with rVSV-∆G-spike resulted in a rapid and potent induction of SARS-CoV-2 neutralizing antibodies. The vaccination protected hamsters against the SARS-CoV-2 challenge, as demonstrated by the prevention of weight loss, and by the alleviation of the extensive tissue damage and viral loads in the lungs and the nasal turbinates (Yahalom-Ronen et al., 2020).

The large-scale use of both hamsters and mice, and the limited indications for disease manifestation or treatment efficacy, required a continued, high-throughput, nonintrusive method for the evaluation of disease appearance and progression, and its alteration by potential vaccines and treatments. We recently introduced a method for monitoring spontaneous group activity of mice in a home cage. Validation of the home cage monitoring system (HCMS100) was based on a disease state induced by the pulmonary exposure to LPS or influenza virus. The overall activity of mice groups tested together demonstrated a clear rate and temporal differences in activity between groups of diseased and control mice over days, in a hands-free and noninvasive fashion (Vagima et al., 2020).

Here, we used the HCMS100 to detect changes in the group activity of both Syrian golden hamsters and K18-hACE2 mice housed in their home cage following SARS-CoV-2 infection. We also studied the effect on group activity following reinfection in hamsters. To further corroborate the rVSV-ΔG-spike vaccination efficacy against SARS-CoV-2, we used the HCMS100 to monitor vaccinated hamster group activity and detected a faster recovery rate. This efficient, nonintrusive, and safe method of monitoring continuous activity, mainly regarding BSL-3 requirements, offers a sensitive measure of disease intensity and progression as well as the vaccine efficacy against SARS-CoV-2 in animal models.

## Results

**FIGURE 1:**
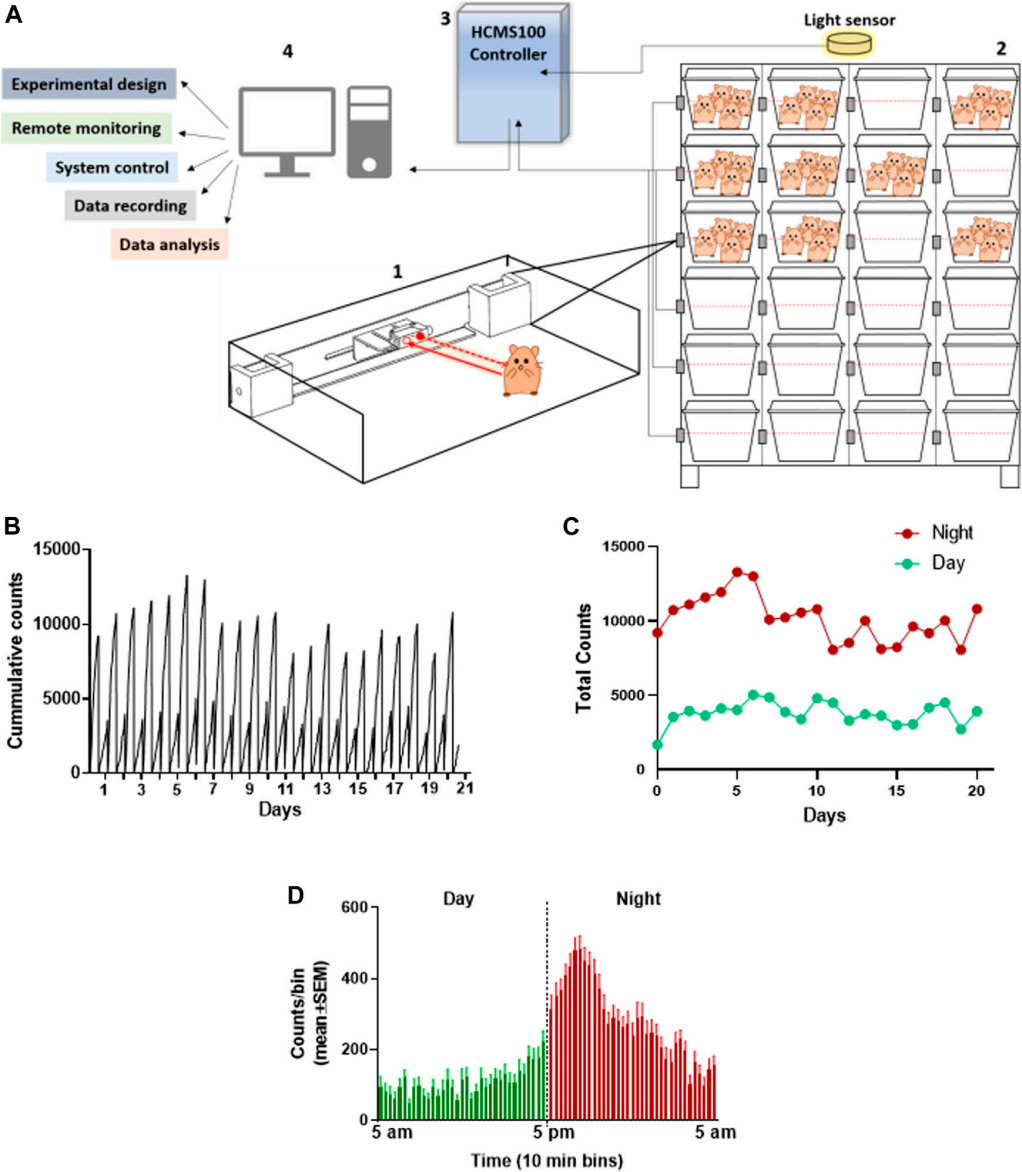
Total activity of 4 Syrian golden hamsters in their home cage recorded continuously over 20 days using the HCMS100. **(A)** HCMS100 schematic outline: A single retroreflective sensor is located externally to each cage at the feeding zone area **(1)**; multiple cages with 4 hamsters/cage are located on a cage rack wired with an individual sensor for each cage **(2)**. The detectors are connected to a communication controller **(3)** with a light sensor to enable data collection and analysis of circadian activity in the cage **(4)**. **(B)** Activity counts of a group of 4 hamsters in a home cage in 10 min bins with lights on from 5 am to 5 pm. Cumulative activity record resets at light change (5 am/5 pm). **(C)** Total counts of activity during nighttime (5 pm–5 am) vs daytime (5 am–5 pm) over 20 days **(D)** Activity counts for the same group of hamsters averaged (±SEM) in 10 min bins over 20 days to demonstrate the overall pattern of nocturnal increase in activity, peaking approximately 1–2 h into the dark cycle.

The group activity of hamsters has been evaluated as a measure of animal’s health. The feasibility of monitoring the baseline activity of a group of hamsters in their home cage was examined by the use of the HCMS100 (Vagima et al., 2020). This system is based on a single laser beam and a detector that horizontally crosses the cage at the feeding zone and animals crossing the laser beam trigger, an event recorder (Figure 1A, Supplement 1). Activity was clearly higher during the night hours than that during daytime in these nocturnal animals as can be seen in the detailed cumulative record of night vs day activity presented in 10-min bins over 20 days. Activity record was reset at light change (5 am and 5 pm, Figure 1B). These data are summarized separately for day and night total activity per 20 days (Figure 1C). The overall pattern of changes in activity is demonstrated in Figure 1D, in which activity was averaged (±SEM) for each 10 min bin over the 20 days recorded. These data revealed a peak activity at approximately 1–2 h into the night cycle. A similar pattern was also observed in hamsters in a single animal testing (Paul et al., 2011) and is different from the two-peak pattern at light shift (day to night and night to day) seen in groups of mice (Vagima et al., 2020).

**FIGURE 2:**
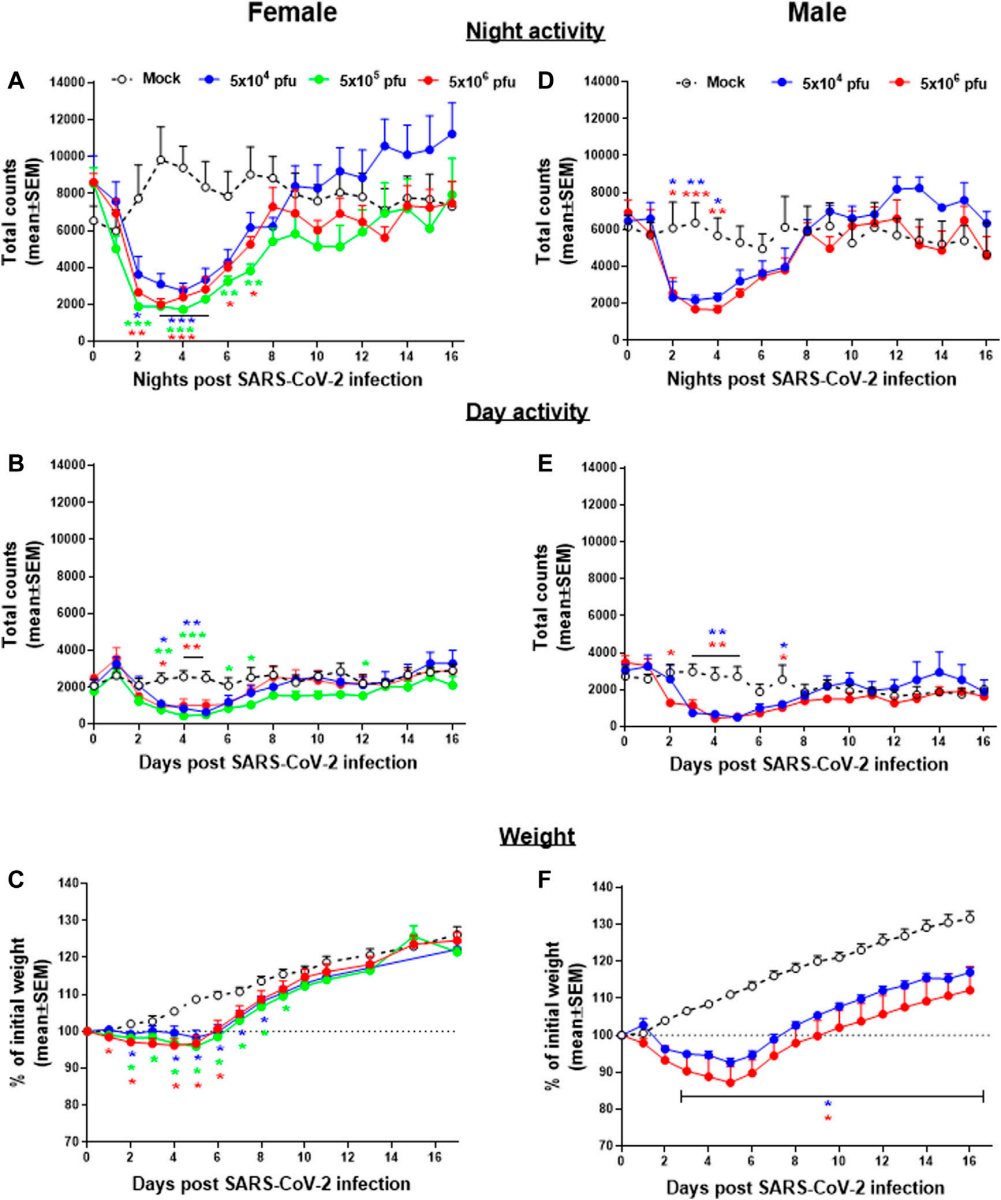
Group activity and body weight changes in SARS-CoV-2–infected hamsters. Total counts of activity during nighttime (5 pm–5 am, **A,D**), daytime (5 am–5 pm, **B,E**), and weight changes **(C,F)** over 16 days of female **(A,B, and C)** and males **(D,E, and F)** hamsters infected i.n. with different doses of SARS-CoV-2. n = 3-5 cages with 4 hamsters/cage. *p < 0.05, **p < 0.01, ***p < 0.001 vs Mock. Asterisks are color coded according to viral load as indicated.

Group activity of male and female hamsters infected intranasally (i.n.) with different doses of SARS-CoV-2 were continuously monitored by the HCMS100 (Figure 2). Control (Mock) females were more active than males during nighttime, with average counts of ∼9,000 counts/12 h compared to ∼5,500 counts/12 h counts, respectively. Two days post SARS-CoV-2 infection, both females and males showed a substantial decrease in activity that returned to normal 8 days postinfection (dpi) (Figures 2A,D). Both females and males showed a similar daytime baseline activity with a similar decrease in activity rates after infection (Figure 2B,E). Reduced activity was accompanied by changes in weight loss (Figure 2C,F) in both males and females. A comparison between the two markers of disease state, namely, activity and weight loss, shows that changes in activity precede the changes in body weight, thus highlighting the importance of activity monitoring to evaluate the COVID-19 disease progression.

**FIGURE 3:**
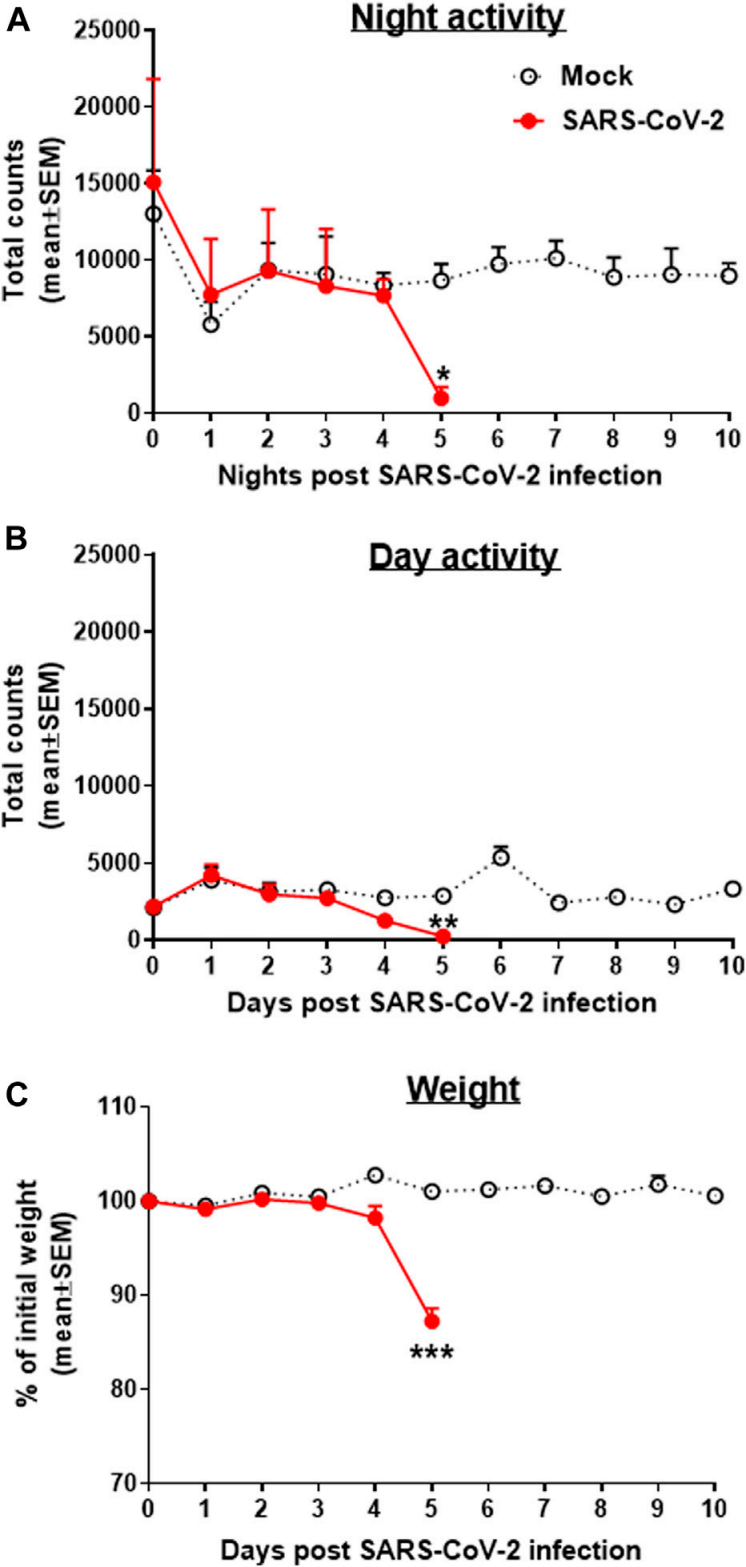
Group activity and weight changes of K18-hACE2 female mice infected with SARS-CoV-2. Total counts of activity during nighttime (5 pm–5 am, **A**) and daytime (5 am–5 pm, **B**) and weight changes **(C)** over 10 days of mice infected i.n. with 2000 pfu of SARS-CoV-2. None of the infected animals survived the infection (death on days 6–7). Infection exposure on day 0, n = 4 cages/group, 5 mice/cage. *p < 0.05, **p < 0.01, ***p < 0.001 vs Mock.

Transgenic mice expressing the human angiotensin I–converting enzyme (ACE2) receptor driven by the cytokeratin-18 gene promoter, K18-hACE2, an additional model for the SARS-CoV-2 infection, were also tested here for changes in group activity following the i.n. infection with a lethal dose of 2000 pfu of SARS-CoV-2 (Figure 3). Infected female mice showed a morbidity effect seen as a sharp decrease in both their night and daytime activities 4–5 days following the SARS-CoV-2 infection, accompanied by reduction in their body weight (Figure 3C). All animals died within 6–7 days following infection.

**FIGURE 4:**
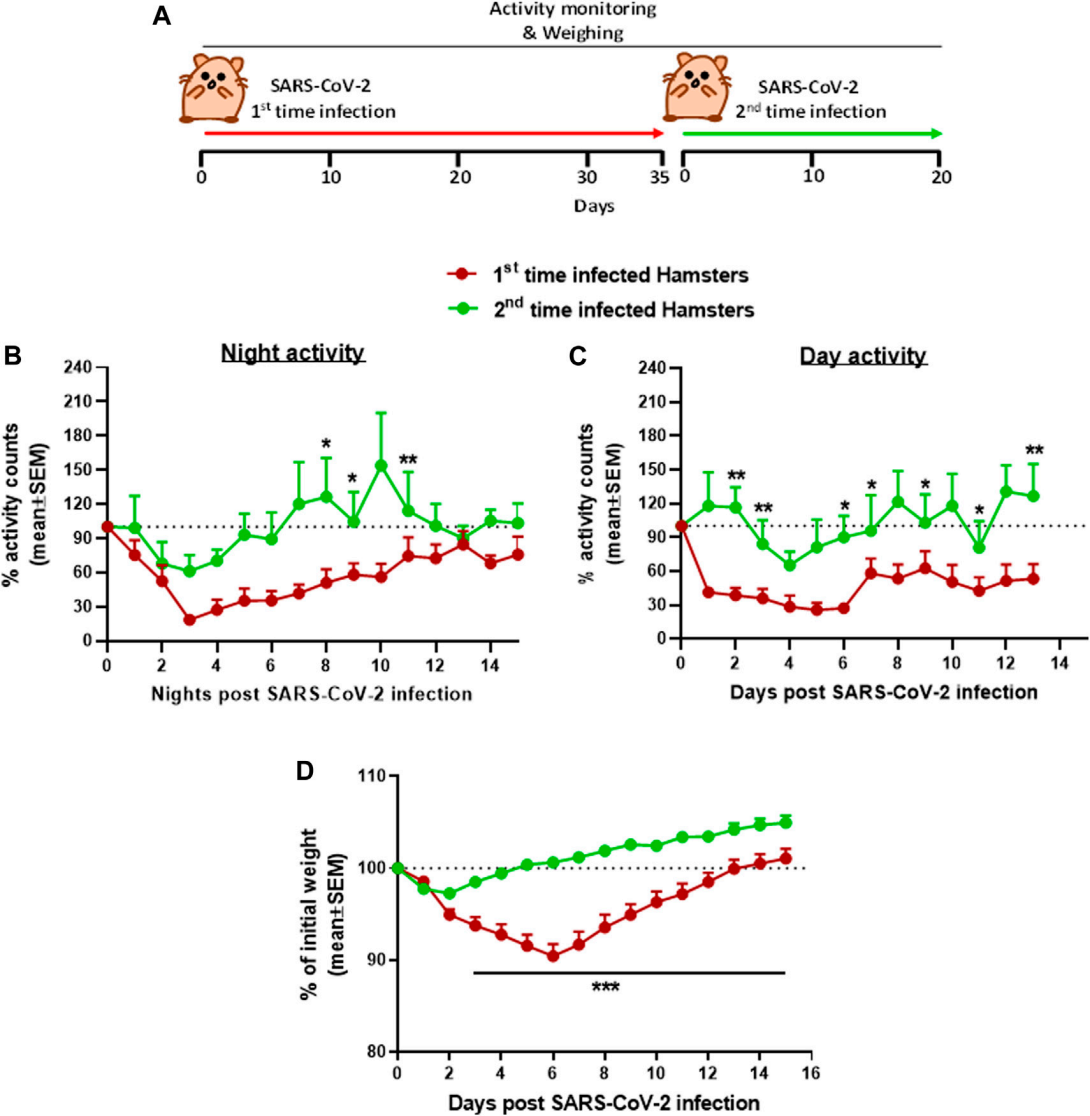
Group activity and body weight changes in male hamsters reinfected with SARS-CoV-2 after recovery. **(A)** Experiment outline: SARS-CoV-2 i.n. infection (5 × 10^6^ pfu/hamster) 35 days after the first infection with the same dose. Percent of total activity counts during nighttime (5 pm–5 am, **B**), daytime (5 am–5 pm, **C**), and weight changes **(D)** over 20 days compared to first time infected. n = 4 same cages of first time and reinfected hamsters, 4 hamsters/cage. *p < 0.05, **p < 0.01, and ***p < 0.001.

The use of hamsters as a model to develop treatment against the SARS-CoV-2 infection was first tested for the effects of re-exposure of convalescent hamsters to SARS-CoV-2 (Figure 4). Male hamsters initially exposed to 5 × 10^6^ pfu of SARS-CoV-2, showed the previously reported decrease in both day and nighttime activities followed by full recovery (see Figure 2). After 35 days following the first exposure, when sufficient SARS-CoV-2 neutralizing antibodies are expected to provide protection and after returning to the baseline activity as previously described (Imai et al., 2020), the hamsters were reinfected with the same dose of 5 × 10^6^ pfu SARS-CoV-2. Disease progression after reinfection was compared to the disease progression that developed after the initial infection. Figure 4 is a summary of the effects following re-exposure of the same animals to SARS-CoV-2, presented as a percent of activity counts. The decrease in activity following the second infection (reinfection) was 30–40% at its peak on day 3 compared to 70–80% prolonged decrease seen at these same hamsters after the first infection.

**FIGURE 5:**
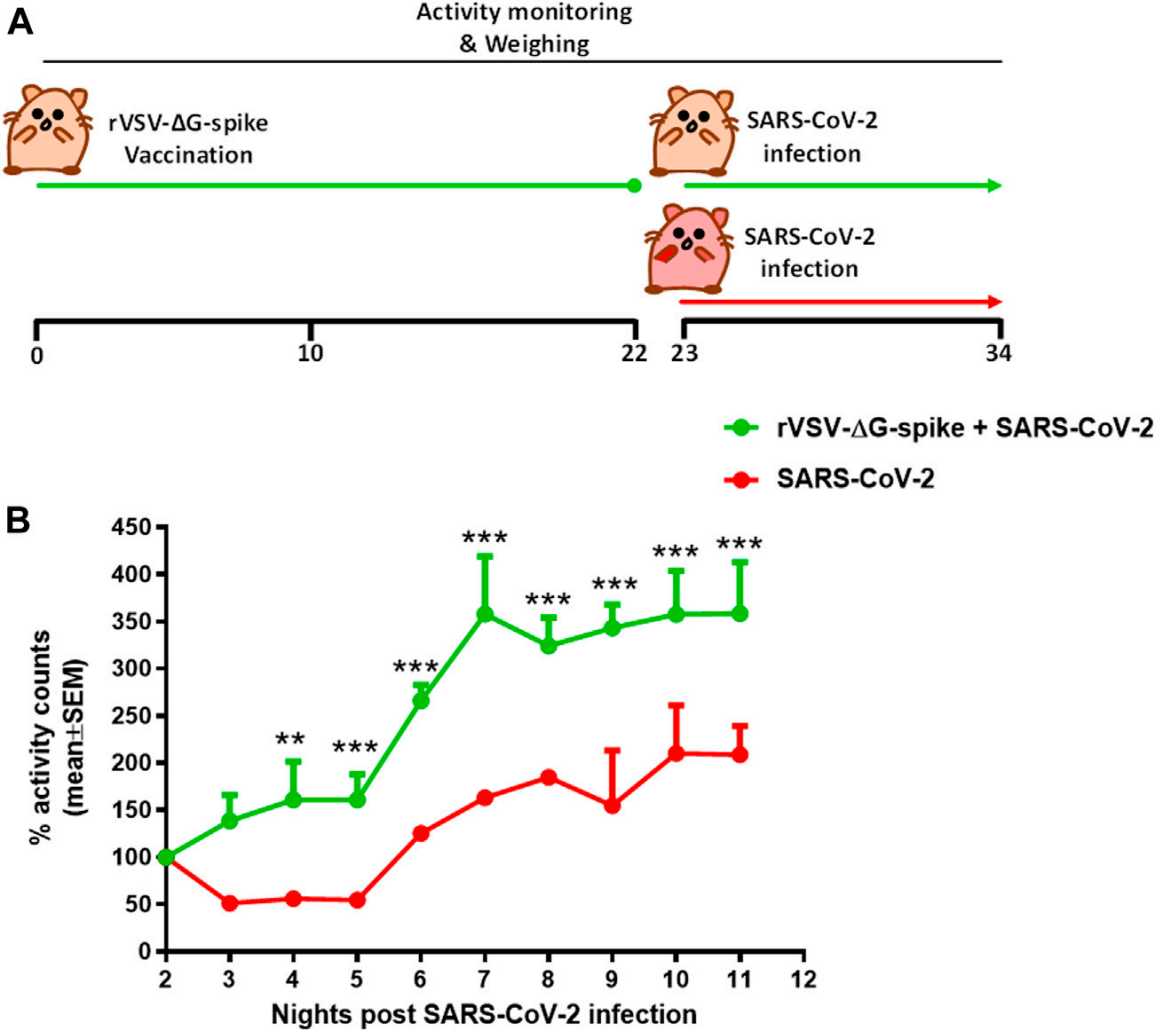
Recovery of group activity in female hamsters vaccinated with rVSV-ΔG-spike following SARS-CoV-2 infection. **(A)** rVSV-ΔG-spike vaccination and SARS-CoV-2 infection scheme. **(B)** Recovery of nighttime group activity of immunized hamsters compared to nonimmunized, two nights after i.n. infection with 5 × 10^6^ pfu of SARS-CoV-2. Recovery was determined as a percent of activity recorded at the disease state 2 days after infection. n = 2 cages/group, 4 hamsters/cage. **p < 0.01 and ***p < 0.001.

Continuous monitoring of group activity was further used to study the efficacy of rVSV-ΔG-spike vaccine as depicted in the scheme (Figure 5A). Hamsters were immunized with 1 × 10^6^ pfu of rVSV-ΔG-spike and monitored for 22 days. We observed normal circadian activity following the vaccination similar to that of naive hamsters (data not shown). On day 23 both immunized and aged matched nonimmunized groups were infected with 5 × 10^6^ pfu of SARS-CoV-2 and the previously reported the nighttime decreased activity was clearly seen. The recovery rate was determined here as a percent of activity at a disease state seen 2 days after infection with 5 × 10^6^ pfu of SARS-CoV-2. Recovery of hamsters pre-immunized with rVSV-ΔG-spike vaccine was seen as a faster and significantly higher group activity compared to nonimmunized hamsters (Figure 5B).

## Materials and Methods

### Cell Lines and Viruses

African green monkey kidney clone E6 cells (Vero E6, ATCC® CRL-1586™) were grown in a Dulbecco’s modified Eagle’s medium (DMEM) containing 10% fetal bovine serum (FBS), MEM nonessential amino acids (NEAA), 2 mM l-glutamine, 100 units/ml penicillin, 0.1 mg/ml streptomycin, 12.5 units/ml nystatin (P/S/N) (Biological Industries, Israel). Cells were cultured at 37°C and 5% CO_2_ at 95% air atmosphere. For hamster’s infection, we used SARS-CoV-2 (GISAID accession EPI_ISL_406862), provided by Bundeswehr Institute of Microbiology, Munich, Germany. For K18-hACE2 transgenic mice infection, we used SARS-CoV-2, isolate Human 2019-nCoV ex China strain BavPat1/2020, that was kindly provided by Prof. Dr. Christian Drosten (Charité, Berlin) through the European Virus Archive Global (EVAg Ref-SKU: 026V-03883). Virus stocks were propagated and tittered on Vero E6 cells. The viruses were stored at -80°C until use. Handling and working with SARS-CoV-2 virus were conducted in a BSL3 facility in accordance with the biosafety guidelines of the Israel Institute for Biological Research (IIBR).

### Animal Experiments

All animal experiments involving SARS-CoV-2 were conducted in a BSL3 facility. The treatment of animals was in accordance with the Animal Welfare Act and the conditions specified in the Guide for Care and Use of Laboratory Animals (National Institute of Health, 2011). Animal studies were approved by the local IIBR ethical committee for animal experiments (protocols numbers HM-01–20, HM-02–20, and M-52–20). Female and male Syrian golden hamsters (60–90 gr., Charles River Laboratories, United States) 6–7 week old and female K18-hACE2 transgenic mice (18–20 gr., Jackson, United States) 8–10 week old were maintained at 20–22°C and a relative humidity of 50±10% on a 12 h light/dark cycle. Animals were fed with commercial rodent chow (Altromin, Germany) and provided with tap water *ad libitum*. Hamsters and mice were randomly assigned to an experiment and kept in groups of 4 and 5, respectively. Both hamsters and mice were acclimated at their home cage for 3–5 days prior to infection to monitor and record the baseline of group activity.

Infection was performed as previously described (Yahalom-Ronen et al., 2020). Briefly, SARS-CoV-2 was diluted in PBS supplemented with 2% FBS (Biological Industries, Israel) and was used to infect anesthetized hamsters (5 × 10^4^, 5 × 10^5^, and 5 × 10^6^ pfu) and mice (2000 pfu) by 50 µl for hamsters and 20 µl for mice by intranasal (i.n.) instillation of viral suspension.

Vaccination with rVSV-ΔG-spike was performed as previously described (Yahalom-Ronen et al., 2020). Briefly, intramuscular (i.m.) 50 µl/animal of rVSV-ΔG-spike (1 × 10^6^ pfu/animal) were administered to Syrian golden hamsters 23 days prior to i.n. infection with SARS-CoV-2.

### Animal Housing and HCMS100 Monitoring

For the home cage monitoring system, HCMS100, we used an industry standard home cage (Tecniplast® 1285L home cage) and a non-limiting industry standard cage rack (Tecniplast® DGM rack). Monitoring group activity of hamsters and mice in a communal home cage by HCMS100 was performed as previously described (Vagima et al., 2020). Briefly, each cage is adjusted with a single retroflective laser sensor (HT3CL1/4P-M8, Leuze electronics, Germany), which comprises emitter and receiver, mounted adjacent to the home cage. Spontaneous movements of the rodents in the cages as indicated by a laser beam crosses situated in the drinking and feeding area are continuously analyzed and recorded. All detectors are connected to the communication controller, and data collection and analyses are operated *via* a remote user interface. The bin duration of laser crosses was recorded every 10 min.

### Statistical Analysis

Group activity counts are presented as means ± SEM. When noted, some group activity data are presented as mean ± SEM of percent of baseline activity of the same group. Weights are presented as mean ± SEM percent change from baseline of the same group. Group differences are analyzed by two-way ANOVA (group x time, with repeated measure on the latter) followed by an appropriate *post hoc* analysis using GraphPad 7. A value of *p* < 0.05 was accepted as statistically significant. A summary of statistical outcome is presented in Supplement 2.

## Discussion

The coronavirus disease 2019 (COVID-19) pandemic caused by SARS-CoV-2 infection has led to substantial unmet need for treatments. The development of such treatments will require testing them in an appropriate COVID-19 animal model. The preclinical research presented here centered on the detection of “disease state” in two animal species that can appropriately model the COVID-19 disease in humans, namely, the Syrian golden hamsters and K18-hACE2 mice (Cohen, 2020). COVID-19 disease in hamsters is often evaluated based on changes in their body weight, but is rarely evaluated by their overt symptoms. Thus, the only disease symptoms observed in SARS-CoV-2–infected hamsters were subtle with mild clinical features (Chan et al., 2020). In this study, we focused on changes in the spontaneous group activity of hamsters in their home cage using the HCMS100, as an effective way to define and characterize changes in hamster’s health. We monitored group activity of hamsters in their home cage and described a consistent pattern of high nighttime activity and low daytime activity of these nocturnal animals with the peak activity detected at about 2 hours into the night (Figure 1). A similar pattern was obtained in hamsters in single animal testing (Paul et al., 2011), and this pattern is different from the two-peak pattern at light shifts seen in groups of mice (Vagima et al., 2020).

Following the intranasal exposure to SARS-CoV-2, we showed a significant decrease in overall group activity of hamsters both at the nighttime active phase and at the daytime rest phase. This decrease started 1–2 days after infection. Male and female hamsters show a similar pattern of disease state although females are generally more active than males during the night. This method of continuous monitoring of group activity was used together with the monitoring of weight loss as markers of disease progression and recovery. Comparisons between these two markers, group activity and weight loss, suggest that the disease state can be detected as early as 48 h postinfection by the decrease in the group activity that precedes the decrease in total body weight (Figure 2). This may be explained by the notion that SARS-CoV-2 infection led to the reduced activity which may in turn decrease food consumption and induced weight loss. Weight loss seemed to be more prominent in males than in females (Yuan et al., 2021), while activity decreased more in females than in males (Schnur and Barela, 1984). These differences are probably the result of gender differences in baseline at this age: females are more active than males and males gain more weight than females. In addition, a few days of weight loss in male following infection seemingly retards full recovery as the control animals continue to gain weight in the normal rate. Although the difference in weight remains statistically significant, the infected animals are fully recovered by days 7–8. Thus, the use of weight loss as the sole marker of the disease may lead to a possible interpretation bias.

K18-hACE2 transgenic mice expressing human angiotensin I–converting enzyme 2 (ACE2) (Yang et al., 2007) were recently established to model human COVID-19 disease and also serve as a reliable model for anti–SARS-CoV-2 neutralizing antibody treatment (Winkler et al., 2020; Rosenfeld et al., 2021; Yinda et al., 2021; Zheng et al., 2021). We observed a sharp decrease in the group activity and weight loss 5 days following viral exposure and death at 6–7 days postinfection. Our data are consistent with the elegant study by Winkler et al., where assessment of COVID-19 disease progression in K18-hACE2 was evaluated using a single animal treadmill stress test (Winkler et al., 2020). However, the use of the HCMS100 presented here eliminated the disruptions in mice habitat and social behavior and the stress associated with it (Borsini et al., 1989). In addition, HCMS100 remote, hands-free method of observation better addressed the safety issues associated with the experimental use of SARS-CoV-2 virus in BSL3 facilities.

Convalescent hamsters previously exposed to SARS-CoV-2 and recovered are immune to SARS-CoV-2 and are expected to be protected from reexposure to the same virus strain (Imai et al., 2020). This was also demonstrated here by monitoring the group activity using the HCMS100 and the body weight changes. After the second exposure to SARS-CoV-2 (35 days after the first exposure), group activity was almost unaffected in convalescent hamsters, compared to the naïve group that exhibited the expected decrease in activity postinfection. This decrease in activity preceded the decrease in weight loss clearly seen in hamsters exposed to the virus for the first time. The weight of the newly infected animals returned to the baseline level at approximately 20 days postinfection, while convalescent hamsters returned to their initial weight 5 days postinfection. (Figure 4D).

Continuous group activity monitoring was further used to study the efficacy of the newly developed rVSV-ΔG-spike vaccine (Yahalom-Ronen et al., 2020). In this pilot study, immunized animals were compared to a group of non-vaccinated hamsters in their response to SARS-CoV-2 infection. Following infection, both groups exhibited a decreased night time activity. However, the group pre-immunized with rVSV-ΔG-spike vaccine showed a shorter morbidity phase as depicted by faster recovery to baseline levels (Figure 5).

The experiments described here demonstrate the importance of the use of behavioral monitoring of infected animals. Applying the home cage monitoring system in studies of disease progression and treatments is on the rise (Voikar and Gaburro, 2020); however, most available monitoring systems are limited to test a single animal per cage which enforces the untoward isolation of the experimental animals. In addition, the relatively high cost of these systems prohibits their use in large-scale experiments commonly used in this type of research. The advantages of the HCMS100 used here were previously detailed (Vagima et al., 2020) and include the continuous monitoring of group activity for as long as required with minimal experimenters interference. This is a clear advantage in experimental designs that require exposure to pathogens such as SARS-CoV-2 that restricts access to the experimental animals.

To date, this HCMS100 monitoring system was successfully used to detect changes in the home cage continuous group activity of mice, hamsters, and rats (unpublished data) with no need of alteration in either hardware or software of the HCMS100. Thus, the system can be easily applied to test additional small animal species in the process of establishing preclinical evaluation of vaccines and drugs for human diseases.

## Data Availability

The original contributions presented in the study are included in the article/Supplementary Material; further inquiries can be directed to the corresponding author.
