# Supplementary material for: Monitoring Group Activity of Hamsters and Mice as a Novel Tool to Evaluate COVID-19 Progression, Convalescence, and rVSV-ΔG-Spike Vaccination Efficacy
Source: Front Bioeng Biotechnol. 2021 Oct 1;9:737627. doi: 10.3389/fbioe.2021.737627 (PMC8517107; doi:10.3389/fbioe.2021.737627)
Supplement: Supplementary file 1 [file DataSheet1.docx]

**Supplementary data**

**Supplement 1: HCMS100 instillation on a standard animal cage rack**


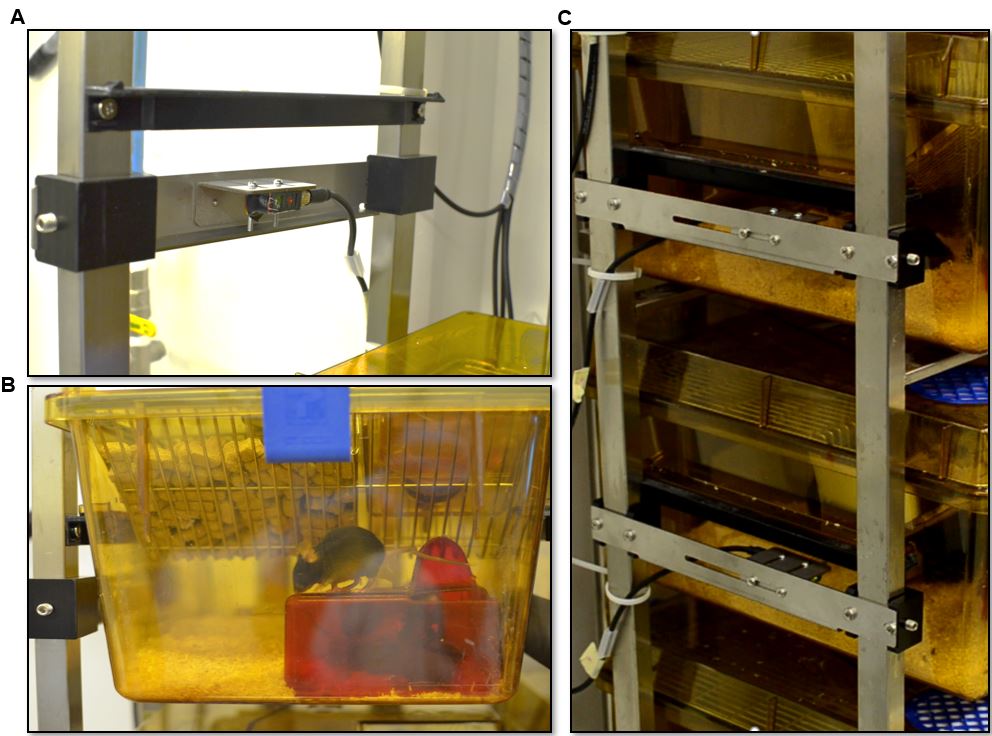


**HCMS100 retrofitted to existing home cages and cage racks.** (A) Mounting assembly comprises backplane, column clamps, and detector mount slidably attached to backplane such that the position of detector mount can be adjusted**.** Detector comprises an emitter and a receiver in a single housing. (B) Detector adjustment can be externally retrofitted to any kind of standard cage and cage rack, of animal usually used in laboratory experimentation, without interfering with cage positioning in its cage rack. (C) Each detector is in wired or wireless data communication with an adjacent controller that can harbor as many detectors (including sensors for temperature, light, humidity, homecage pH, food level, water level, animal play devices and so forth), and is in wired or wireless data communication with a remote analyzer.

**Supplement 2: Significant outcome of the statistical analyses**

**Figure 2**

**2 ways ANOVA with repeated measure (dose x time)**

|  |  | **Female** | **Male** |
| --- | --- | --- | --- |
| **Night time activity** | **Interaction** | **F (48, 272) = 5.281, p<.0001** | **F(30,135)=4.45, p<.0001** |
|  | **Time** | **F (16, 272) = 18.85, p<.0001** | **F(15,135)=13.84, p<.0001** |
|  | **Dose** | **ns** | **ns** |
|  | **Post hoc Dunnett test** | **p<.04-.0001** | **p<.02-.0003** |
| **Day time activity** | **Interaction** | **F(48,272)=1.883, p=.0009** | **F (32, 144) = 3.642, p<.0001** |
|  | **Time** | **F(16,272)=16.59, p<.0001** | **F (16, 144) = 8.378, p<.0001** |
|  | **Dose** | **ns** | **ns** |
|  | **Post hoc Dunnett test** | **p<.02-.0001** | **p<.04-.0001** |
| **Weights** | **Interaction** | **F(42,672)=3.97, p<.0001** | **F (40, 440) = 15.16, p<.0001** |
|  | **Time** | **F(14,672)=387, p<.0001** | **F (20, 220) = 692.4, p<.0001** |
|  | **Dose** | **F(3,48)4.38, p=.0084** | **F (2, 22) = 47.85, p<.0001** |
|  | **Post hoc Dunnett test** | **p<.05-.0001** | **P< .0001** |

**Figure 3**

**2 ways ANOVA with repeated measure (dose x time)**

| **Night time activity** | **Interaction** | **F (5, 25) = 4.232, p=.0063** |
| --- | --- | --- |
|  | **Time** | **F (5, 25) = 12.81, p<.0001** |
|  | **Dose** | **ns** |
|  | **Post hoc Dunnett test** | **ns** |
| **Day time activity** | **Interaction** | **F (5, 30) = 5.624, p<.0009** |
|  | **Time** | **F (5, 30) = 14.84, p<.0001** |
|  | **Dose** | **ns** |
|  | **Post hoc Dunnett test** | **P=.0024** |
| **Weights** | **Interaction** | **F (5, 190) = 51.2, p<.0001** |
|  | **Time** | **F (5, 190) = 43.18, p<.0001** |
|  | **Dose** | **F (1, 38) = 31.34, p<.0001** |
|  | **Post hoc Dunnett test** | **P< .0001** |

**Figure 4**

**2 ways ANOVA with repeated measure (dose x time)**

| **Night time activity** | **Interaction** | **F (15, 90) = 1.98, p=.0253** |
| --- | --- | --- |
|  | **Time** | **F (15, 90) = 3.85, p<.0001** |
|  | **Dose** | **ns** |
|  | **Post hoc 2-stage linear step-up procedure of Benjamini, Krieger and Yekutieli** | **p=.01-.004** |
| **Day time activity** | **Interaction** | **ns** |
|  | **Time** | **F (13, 78) = 3.02, p=.0012** |
|  | **Dose** | **F (1, 6) = 8.49, p=.0268** |
|  | **Post hoc 2-stage linear step-up procedure of Benjamini, Krieger and Yekutieli** | **P=.01-.03** |
| **Weights** | **Interaction** | **F (15, 450) = 33.37, p<.0001** |
|  | **Time** | **F (15, 450) = 87.59, p<.0001** |
|  | **Dose** | **F (1, 30) = 27.83, p<.0001** |
|  | **Post hoc 2-stage linear step-up procedure of Benjamini, Krieger and Yekutieli** | **P< .01-.0001** |

**Figure 5**

**2 ways ANOVA with repeated measure (dose x time)**

| **% night time activity** | **Interaction** | **F (9, 9) = 3.709, p=.03** |
| --- | --- | --- |
|  | **Time** | **F (9, 9) = 17.59, p=.0001** |
|  | **Dose** | **ns** |
|  | **Post hoc 2-stage linear step-up procedure of Benjamini, Krieger and Yekutieli** | **P<.01-.0006** |
